# VALIDATING THE IMPACT OF THE COMPREHENSIVE MOBILITY EVALUATION TOOL USED BY NURSES IN ACUTE CARE

**DOI:** 10.1093/geroni/igad104.0192

**Published:** 2023-12-21

**Authors:** Mary Hook

**Affiliations:** Advocate Health, Milwaukee, Wisconsin, United States

## Abstract

Existing nursing-based mobility tools were developed with clinical and reliability testing with small samples; none focused on predicting assistance needs. The Comprehensive Mobility Evaluation Tool (CMET) was designed using established testing procedures to support nurses to assess all phases of mobilization and predict level of assistance. A prospective descriptive research design was used to test reliability and validate CMET items, scoring, and ability to predict LOA. A randomly selected sample of decisional/English-speaking non-ventilated stable patients were enrolled from four medical/surgical and one critical care units at a large community hospital where CMET was used. Trained data collectors consented patients, completed bedside testing, and distributed patient surveys. Descriptive, inferential, and regression statistics were used for analysis. The representative sample (N=190) had an average age of 67.3 yrs (SD=16.2, 23-101), 50% male, with a robust range of mobility levels. Inter-rater reliability (n=27) yielded an average kappa of 0.94. Six of seven steps contributed significantly to predicting patient LOA with scores that correlated with nurse judgement [r (184) =0.86, p< .0001]. Patients needed time and support to perform testing without assistance for optimal accuracy and benefit. Patients reported CMET was useful (88%) and easy (62%) and identified barriers that limit walking while hospitalized. Study limited by research at a single site. This study represents the initial validation, confirming reliability and benefits of the CMET, a skill-based tool to evaluate mobility status. Providing time and encouragement to perform CMET helps nurses and patients to identify deficits and tailor care to overcome mobility barriers.

